# Co-exposure to multi-walled carbon nanotube and lead ions aggravates hepatotoxicity of nonalcoholic fatty liver via inhibiting AMPK/PPARγ pathway

**DOI:** 10.18632/aging.103430

**Published:** 2020-07-17

**Authors:** Enqin Liu, Xinghui Wang, Xidong Li, Ping Tian, Hao Xu, Zenglian Li, Likun Wang

**Affiliations:** 1 Department of Infectious Diseases, Linyi People's Hospital, Linyi, China; 2 Department of Respiratory Medicine, Affiliated Hospital of Shandong Medical College, Linyi, China

**Keywords:** multi-walled carbon nanotube, lead ions, NAFLD, AMPK, PPARγ

## Abstract

Multi-walled carbon nanotubes (MWCNTs) have been widely used in sewage disposal, water purification, and disinfection. Co-exposure to MWCNTs and heavy metal ions is common during water disposal. However, the hepatotoxicity of co-exposure to MWCNTs and lead ions for nonalcoholic fatty liver disease (NAFLD) subjects has not been investigated. NAFLD mice were fed intragastrically with MWCNTs and lead acetate (PbAc). Combined administration of MWCNTs and PbAc significantly damaged the liver function, and aggravated the nonalcoholic steatohepatitis phenotype as well as the hepatic fibrosis and steatosis in NAFLD mice. Furthermore, MWCNTs and PbAc significantly induced apoptosis in primary hepatocytes isolated from NAFLD mice. Combined administration of MWCNTs and PbAc also resulted in hepatic lipid peroxidation by inducing antioxidant defense system dysfunction, and significantly enhanced the expression levels of inflammatory cytokines in NAFLD mice livers. Meanwhile, combined administration of MWCNTs and PbAc may exert its hepatotoxicity in the NAFLD via inhibiting the adenosine 5‘-monophosphate activated protein kinase (AMPK)/peroxisome proliferator-activated receptors γ (PPARγ) pathway. Taken together, we conclude that co-exposure to MWCNTs and PbAc can remarkably aggravate the hepatotoxicity in NAFLD mice via inhibiting the AMPK/PPARγ pathway. This study may provide a biosafety evaluation for the application of nanomaterials in wastewater treatment.

## INTRODUCTION

Obesity has become an increasingly prevalent global health problem. According to the data of the World Health Organization, up to 75% - 100% of obese people suffer from nonalcoholic fatty liver disease (NAFLD) [1, 2]. NAFLD is a common hepatic metabolic syndrome, and around 25% of patients will develop non-alcoholic steatohepatitis (NASH). Moreover, a portion of patients will further deteriorate and develop liver fibrosis, cirrhosis, and even liver cancer [3]. As one of the most common chronic metabolic diseases accompanying obesity, the incidence of NAFLD has also increased in the past decades. Environmental factors are believed to be one of the major contributors for NAFLD [4].

Lead ions are common pollutants in the environment. Industrial exhaust gas, sewage and automobile exhaust gas contain a lot of lead ions. It is found that lead ions can cause physiological disorders in the human body, leading to serious damage to liver, kidney and hematopoietic systems [5–7].

Due to their unique physical and chemical properties, multi-walled carbon nanotubes (MWCNTs) are widely used in sewage disposal, water purification, and disinfection [8, 9]. MWCNTs can inevitably absorb lead ions and other pollutants in the water during the process of water treatment, which will cause the organisms to be co-exposed to MWCNTs and lead ions [10, 11]. However, the influence of co-exposure to MWCNTs and lead ions on nonalcoholic fatty liver has not been investigated.

Peroxisome proliferator-activated receptors (PPAR) belong to the nuclear hormone receptor superfamily, and are involved in glucose and fatty acid metabolism via regulating the fatty acid oxidation related genes, such as medium-chain acyl-CoA dehydrogenase and carnitine palmitoyl transferase-1 [12, 13]. Adenosine 5‘-monophosphate activated protein kinase (AMPK) can mediate the metabolic stress and energy homeostasis through controlling several homeostatic mechanisms. Activated AMPK can inhibit the fatty acid synthesis through suppressing acetyl-CoA carboxylase and phosphorylating [14]. It remains unclear whether co-exposure to MWCNTs and lead ions will influence the AMPK/PPAR-γ pathway.

Thus far, the toxicity of co-exposure to MWCNTs and heavy metal ions has only been studied *in vitro*. In the present study, we successfully constructed a mouse model of nonalcoholic fatty liver. Meanwhile, we systematically evaluated the hepatotoxicity of MWCNTs and lead ions to the healthy and NAFLD mice, and the potential mechanisms were firstly reported. Our research could provide a biosafety evaluation for the application of nanomaterials in wastewater treatment.

## RESULTS

### NAFLD models were successfully established in mice

**Figure 1:**
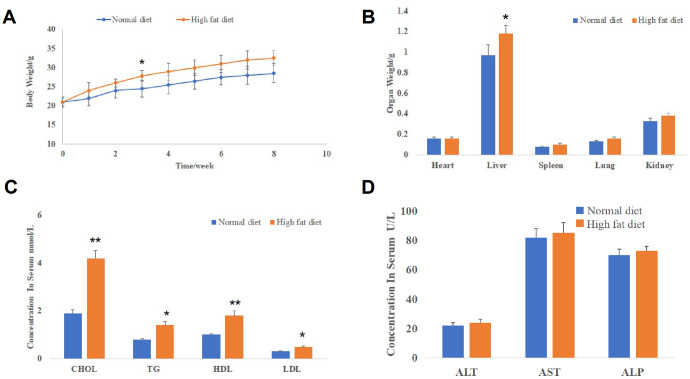
**NAFLD models were successfully established in mice.** (**A**) The body weight change curve of mice in the normal diet and high-fat diet groups (*P<0.05, compared to normal diet mice). (**B**) The weight of mice organs including the liver, heart, lung, spleen and kidney in the normal diet and high-fat diet groups (*P<0.05, compared to normal diet mice). (**C**) The serum levels of lipoproteins (CHOL, TG, HDL and LDL) of mice in the normal diet and high-fat diet groups (*P<0.05 and **P<0.01, compared to normal diet mice). (**D**) The serum levels of hepatic damage biomarkers (ALT, AST and ALP) in the normal diet and high-fat diet groups.

As shown in Figure 1A, the body weight of mice in both groups was increased gradually over time. Meanwhile, we found that the body weight of mice in the high-fat diet group was significantly higher than that of mice in the normal diet group since the 3^th^ week. Furthermore, the liver weight of mice in the high-fat diet group was significantly higher than that in the normal diet group, while the weight of heart, lung, spleen and kidney had no significant difference between the two groups (Figure 1B). Further results showed that the serum levels of four lipoproteins (cholesterol (CHOL), triglyceride (TG), high-density lipoprotein (HDL), low-density lipoprotein (LDL)) of mice in the high-fat diet group were significantly increased compared to that in the normal diet group (Figure 1C). Finally, we detected that the high-fat diet had no significant effects on the serum levels of hepatic damage biomarkers (alanine aminotransferase (ALT), aspartate aminotransferase (AST) and alkaline phosphatase (ALP); Figure 1D). These data indicated that NAFLD models were successfully established in mice and there were no obvious hepatic damages in NAFLD mice.

### MWCNTs and PbAc exposure significantly reduced the body weight of mice

**Figure 2:**
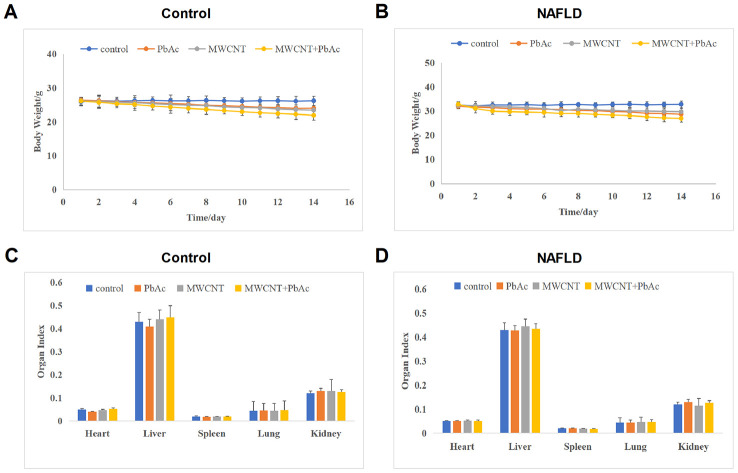
**MWCNTs and PbAc exposure significantly reduced the body weight of mice.** (**A**, **B**) The body weight change curve of the control and NAFLD mice upon the low dose of PbAc, MWCNTs or MWCNTs + PbAc administration. (**C**, **D**) The impacts of the low dose of PbAc, MWCNTs or MWCNTs + PbAc on the organ index of liver, heart, lung, spleen and kidney of both the control and NAFLD mice.

We found that the high dose of PbAc, MWCNTs or MWCNTs + PbAc could lead to death in both the control and NAFLD mice. Therein, the high dose of MWCNTs + PbAc could cause mouse death on the 5^th^ day and the final survival rate was less than 20% (Supplementary Figure 2A, 2B). While no mouse death was observed in low dose groups. Therefore, subsequent data collection and analysis in our study were performed on mice in low dose groups. Firstly, we detected that the low dose of PbAc, MWCNTs or MWCNTs + PbAc could lead to significant body weight reduction in both the control and NAFLD mice. Moreover, we found that combined administration of MWCNTs and PbAc induced more obvious decreases in the body weight of mice than single administration of PbAc or MWCNTs (Figure 2A, 2B). Secondary, we found that the low dose of PbAc, MWCNTs or MWCNTs + PbAc had no significant influences on the organ index of liver, heart, lung, spleen and kidney of both the control and NAFLD mice (Figure 2C, 2D).

### Combined administration of MWCNTs and PbAc significantly damaged the liver function in NAFLD mice

**Figure 3:**
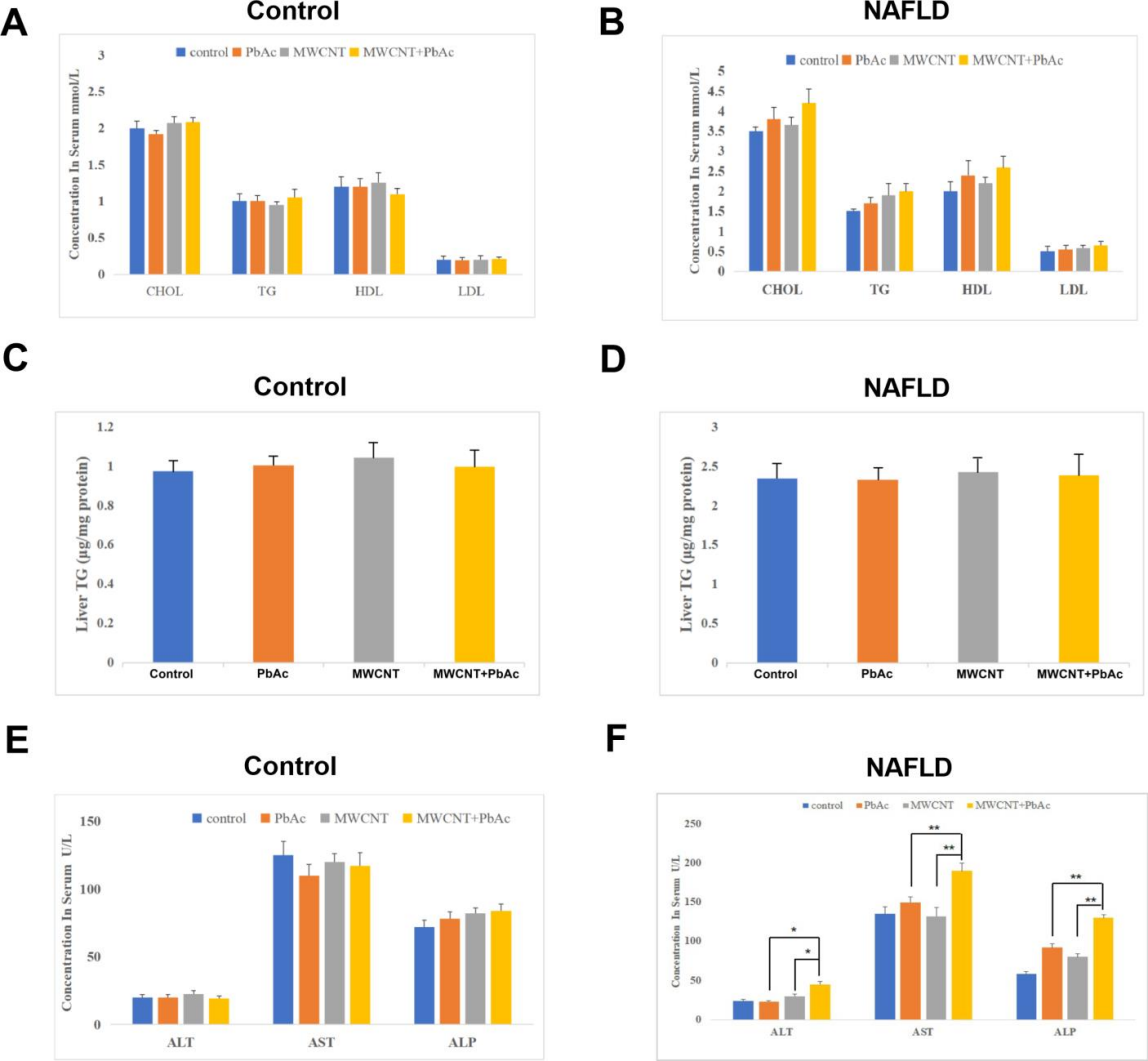
**Combined administration of MWCNTs and PbAc significantly damaged the liver function in NAFLD mice.** The effects of low dose of PbAc, MWCNTs or MWCNTs + PbAc on the serum levels of CHOL, TG, HDL, LDL (**A**, **B**) and the TG expression levels in liver tissues (**C**, **D**) in the control and NAFLD mice. (**E**, **F**) Changes in the serum levels of ALT, AST and ALP in the control and NAFLD mice upon the low dose of PbAc, MWCNTs or MWCNTs + PbAc administration (*P<0.05 and **P<0.01, compared to PbAc or MWCNTs).

We tried to further explore the effects of MWCNTs and PbAc exposure on the serum levels of lipoproteins and hepatic damage biomarkers in mice. We found that upon the low dose of PbAc, MWCNTs or MWCNTs + PbAc administration, the serum levels of CHOL, TG, HDL, LDL and the TG expression levels in liver tissues were not significantly altered in both the control and NAFLD mice (Figure 3A–3D). Furthermore, we detected that the low dose of PbAc, MWCNTs or MWCNTs + PbAc did not significantly change the serum levels of ALT, AST and ALP in control mice (Figure 3E), while combined administration of MWCNTs and PbAc resulted in more significant decreases in the serum levels of ALT, AST and ALP than single administration of PbAc or MWCNTs in NAFLD mice (Figure 3F), indicating that combined administration of MWCNTs and PbAc significantly damaged the liver function in NAFLD mice.

### MWCNTs and PbAc exposure significantly aggravated the nonalcoholic steatohepatitis phenotype in NAFLD mice

**Figure 4:**
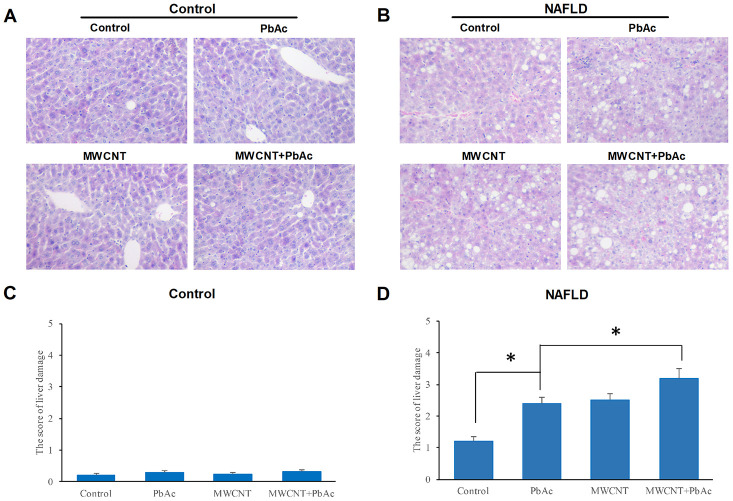
**MWCNTs and PbAc exposure significantly aggravated the nonalcoholic steatohepatitis phenotype in NAFLD mice.** (**A**, **B**) Histological morphology changes in livers of the control and NAFLD mice exposed to the low dose of PbAc, MWCNTs or MWCNTs + PbAc administration. (**C**, **D**) The score of liver damage in the control and NAFLD mice upon the low dose of PbAc, MWCNTs or MWCNTs + PbAc administration (*P<0.05 and **P<0.01, compared to saline water (control) or PbAc).

We performed H&E staining to test the hepatic histological alterations ascribed to MWCNTs and PbAc exposure in control and NAFLD mice. We found that the low dose of PbAc, MWCNTs or MWCNTs + PbAc did not obviously damage the livers of control mice (Figure 4A, 4C), while the manifestations of hepatic steatosis and lobular inflammation were remarkably aggravated due to the low dose of PbAc, MWCNTs or MWCNTs + PbAc administration in NAFLD mice compared to the saline water (Figure 4B). Results of liver damage scoring showed that hepatic damages induced by co-exposure to MWCNTs and PbAc were the most prominent (Figure 4D).

### MWCNTs and PbAc exposure significantly aggravated the hepatic fibrosis and steatosis in NAFLD mice

**Figure 5:**
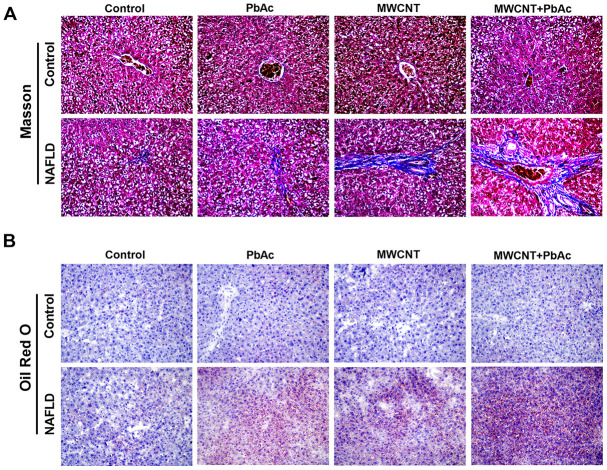
**MWCNTs and PbAc exposure significantly aggravated the hepatic fibrosis and steatosis in NAFLD mice.** (**A**) Upon the low dose of PbAc, MWCNTs or MWCNTs + PbAc administration, Masson staining was conducted to detect the collagen deposition (blue indicates collagen) in liver tissues of control and NAFLD mice. (**B**) Oil red O staining was performed to detect the lipidoses (red indicates lipid) in liver tissues of control and NAFLD mice.

To further research the hepatotoxic effects of MWCNT and PbAc exposure, we conducted Masson staining and Oil red O staining to detect the collagen deposition and lipidoses in liver tissues, respectively. Results demonstrated that the low dose of PbAc, MWCNTs or MWCNTs + PbAc induced obvious collagen deposition and lipidoses in liver tissues of NAFLD mice, while these two phenomena were not observed in control mice. Meanwhile, we also found that combined administration of MWCNTs and PbAc caused more remarkable collagen deposition and lipidoses than single administration of MWCNTs and PbAc in NAFLD mice (Figure 5A, 5B). These results suggested that MWCNTs and PbAc exposure significantly aggravated the hepatic fibrosis and steatosis in NAFLD mice.

### MWCNTs and PbAc exposure significantly induced apoptosis in primary hepatocytes isolated from NAFLD mice

**Figure 6:**
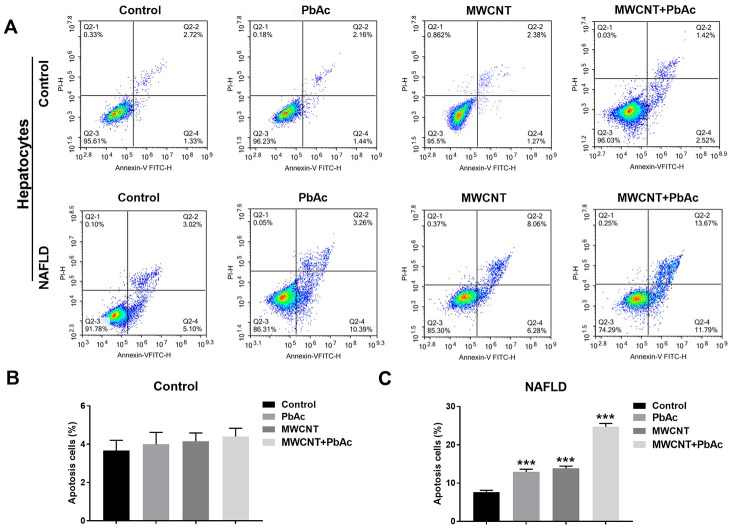
**MWCNTs and PbAc exposure significantly induced apoptosis in primary hepatocytes isolated from NAFLD mice.** (**A**) After treatment with the low dose of PbAc, MWCNTs or MWCNTs + PbAc, apoptotic analyses were performed in primary hepatocytes from control and NAFLD mice using a flow cytometry. (**B**, **C**) Apoptotic rates in primary hepatocytes from control and NAFLD mice were statistically analyzed (***P<0.001, compared to saline water).

We isolated primary hepatocytes from control and NAFLD mice to further investigate the hepatoxicity of MWCNTs and PbAc exposure. As shown in Figure 6A, apoptotic analyses were performed in primary hepatocytes using a flow cytometry. We found that the low dose of PbAc, MWCNTs or MWCNTs + PbAc did not significantly alter the proportion of apoptotic cells in primary hepatocytes from control mice (Figure 6B), but significantly increased the apoptotic rates in primary hepatocytes from NAFLD mice, and such increase caused by co-exposure to MWCNTs and PbAc was the most prominent (Figure 6C).

### Combined administration of MWCNTs and PbAc resulted in hepatic lipid peroxidation by inducing antioxidant defense system dysfunction

**Figure 7:**
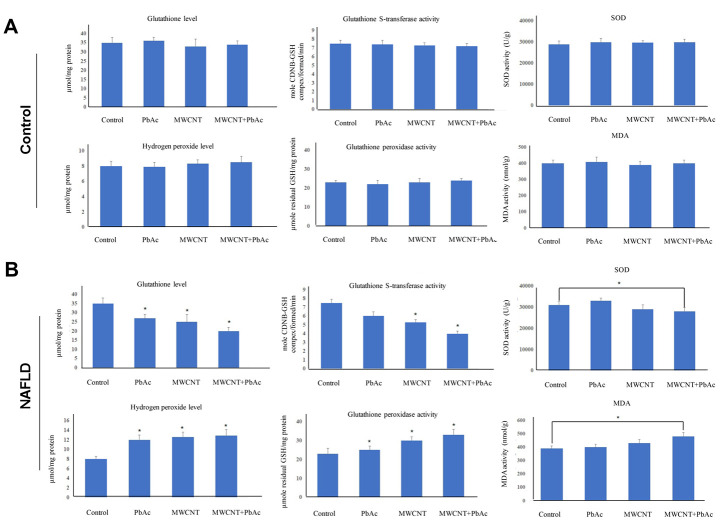
**Combined administration of MWCNTs and PbAc resulted in hepatic lipid peroxidation by inducing antioxidant defense system dysfunction.** (**A**, **B**) Alterations in SOD, GST, GPx activities as well as GSH, hydrogen peroxide, MDA levels in the control and NAFLD mice livers exposed to the low dose of PbAc, MWCNTs or MWCNTs + PbAc administration (*P<0.05, compared to saline water).

Next, we tested the hepatic redox status in mice upon the low dose of PbAc, MWCNTs or MWCNTs + PbAc administration. Figure 7A depicts that the low dose of PbAc, MWCNTs or MWCNTs + PbAc had no significant effects on hepatic redox status in control mice. Moreover, we detected that combined administration of MWCNTs and PbAc significantly decreased the superoxide dismutase (SOD) and glutathione-S-transferase (GST) activities as well as glutathione (GSH) level in NAFLD mice compared to saline water or single administration of PbAc or MWCNTs. Conversely, we found that combined administration of MWCNTs and PbAc significant increased the hepatic hydrogen peroxide (H_2_O_2_) level and glutathione peroxidase (GPx) activity in NAFLD mice compared to saline water or single administration of PbAc or MWCNTs. Furthermore, we found that there was a significant increase in the hepatic level of malondialdehyde (MDA, a biomarker of lipid peroxidation) in NAFLD mice upon the combined administration of MWCNTs and PbAc (Figure 7B).

### Combined administration of MWCNTs and PbAc significantly enhanced inflammatory cytokine expressions in NAFLD mice livers

**Figure 8:**
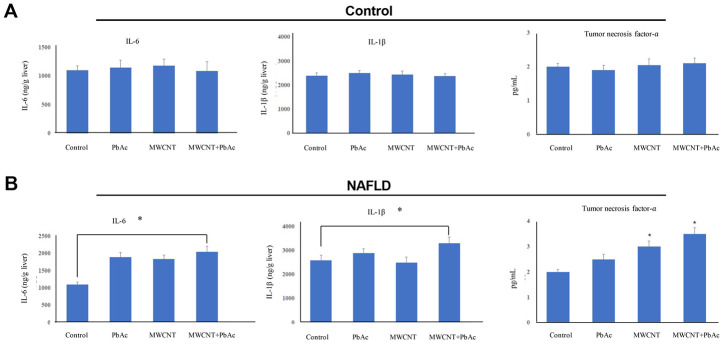
**Combined administration of MWCNTs and PbAc significantly enhanced inflammatory cytokine expressions in NAFLD mice livers.** (**A**, **B**) The effects of the low dose of PbAc, MWCNTs or MWCNTs + PbAc on the expressions of IL-6, IL-1β and TNF-α in the control and NAFLD mice livers (*P<0.05, compared to saline water).

In addition, we found that the low dose of PbAc, MWCNTs or MWCNTs + PbAc had no significant effects on the expressions of inflammatory cytokines namely IL-6, IL-1β and TNF-α in control mice livers (Figure 8A). Meanwhile, we detected that combined administration of MWCNTs and PbAc significantly enhanced the IL-6, IL-1β and TNF-α expressions in NAFLD mice livers compared to saline water or single administration of PbAc or MWCNTs (Figure 8B).

### Combined administration of MWCNTs and PbAc may exert its hepatotoxicity to NAFLD mice via inhibiting AMPK/PPARγ pathway

**Figure 9:**
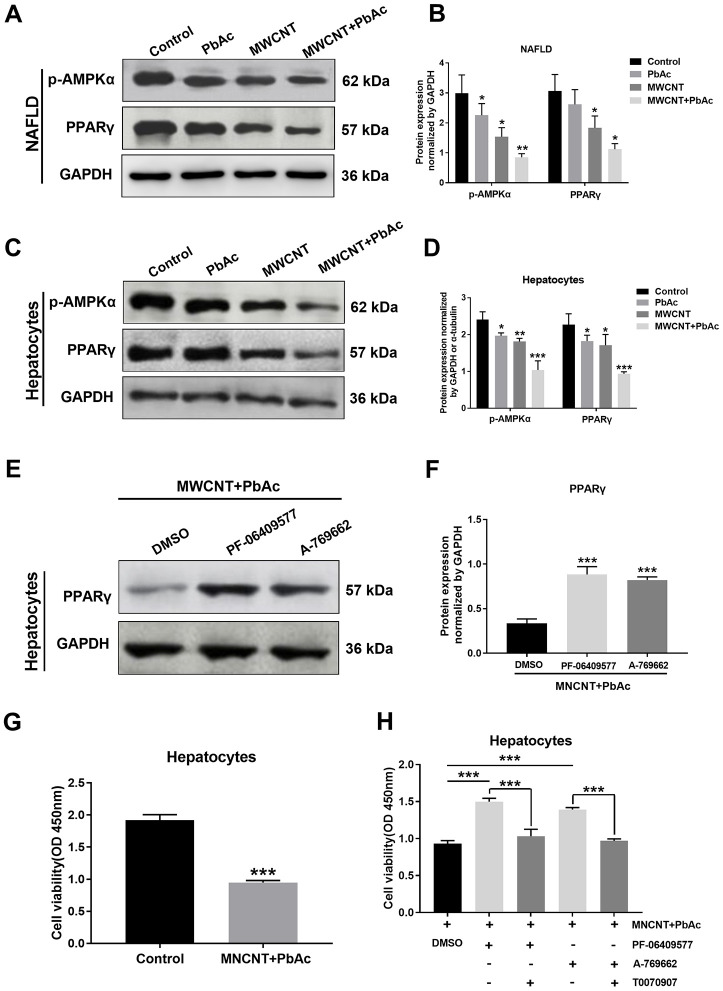
**Combined administration of MWCNTs and PbAc may exert its hepatotoxicity to NAFLD mice via inhibiting AMPK/PPARγ pathway.** Western blot analysis of PPARγ and p-AMPKα expressions in NAFLD mice livers (**A**) and in primary hepatocytes from NAFLD mice (**C**) upon the low dose of PbAc, MWCNTs or MWCNTs + PbAc administration. (**B**, **D**) PPARγ and p-AMPKα expression levels normalized to GAPDH (*P<0.05, **P<0.01 and ***P<0.001, compared to saline water). (**E**) After treatments with DMSO or two AMPK activators (0.5 μM PF-06409577 and 10 μM A-769662) in addition to MWCNTs + PbAc, PPARγ expressions in primary hepatocytes from NAFLD mice were tested using western blot analysis. (**F**) PPARγ expression levels normalized to GAPDH (***P<0.001, compared to DMSO). (**G**) Cell viability of primary hepatocytes from NAFLD mice upon the administration of saline water or MWCNTs + PbAc (***P<0.001, compared to saline water). (**H**) In addition to MWCNTs + PbAc, primary hepatocytes from NAFLD mice were also incubated with DMSO, PF-06409577 (0.5 μM), A-769662 (10 μM) or T0070907 (a selective PPARγ antagonist, 50 μM), then the cell viability was measured (***P<0.001, compared to DMSO or T0070907).

Using western blot analysis, we found that the low dose of MWCNTs or MWCNTs + PbAc significantly decreased the expression levels of p-AMPKα and PPARγ in the livers of NAFLD mice compared to the saline water or single administration of PbAc, and such decreases induced by combined administration of MWCNTs and PbAc were more prominent (Figure 9A, 9B). Furthermore, we obtained the similar results in primary hepatocytes from NAFLD mice (Figure 9C, 9D). After co-treatment with MWCNTs and PbAc, we detected that both the two AMPK activators (PF-06409577 and A-769662) could significantly reverse the PPARγ expression levels in primary hepatocytes from NAFLD mice (Figure 9E, 9F). Finally, we found that combined administration of MWCNTs and PbAc significantly reduced the cell viability of primary hepatocytes from NAFLD mice (Figure 9G). On the contrary, PF-06409577 and A-769662 could significantly reverse the inhibitory effects of co-treatment with MWCNTs and PbAc on primary hepatocytes from NAFLD mice, while a selective PPAR-γ antagonist (T0070907) could significantly eliminate such reverse effects of PF-06409577 and A-769662 (Figure 9H).

**Figure 10:**
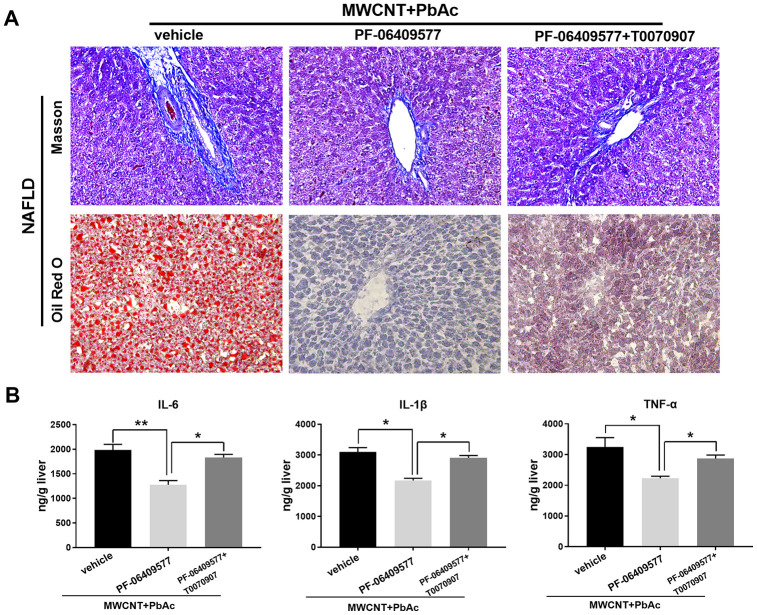
**Intervening AMPK/PPARγ pathway significantly changed the hepatotoxicity of MWCNTs + PbAc to NAFLD mice.** (**A**) Upon the administration of MWCNTs + PbAc, NAFLD mice were simultaneously treated with vehicle, PF-06409577 and PF-06409577 + T0070907. Masson staining and Oil red O staining were performed to detect the hepatic fibrosis (blue indicates collagen) and steatosis (red indicates lipid) in NAFLD mice, respectively. (**B**) The expression levels of IL-6, IL-1β and TNF-α in NAFLD mice livers were measured (**P<0.01 and *P<0.05, compared to PF-06409577).

Moreover, results in Masson staining and Oil red O staining demonstrated that PF-06409577 significantly ameliorated the hepatic fibrosis and steatosis induced by co-treatment with MWCNTs and PbAc in NAFLD mice, while T0070907 significantly attenuated such effects of PF-06409577 (Figure 10A). In addition, we detected that PF-06409577 significantly eliminated the increases in the expression levels of IL-6, IL-1β and TNF-α induced by co-treatment with MWCNTs and PbAc in NAFLD mice livers. Likewise, we found that T0070907 significantly reverse such effects of PF-06409577 (Figure 10B). Taken together, above results indicated that combined administration of MWCNTs and PbAc may exert its hepatotoxicity to NAFLD mice via inhibiting AMPK/PPARγ pathway.

## DISCUSSION

MWCNTs have been widely used in various fields including daily consumable products, agriculture, engineering, nanoelectronics and medicine [15]. Excessive exposure to them has been proved to be a risk to human health [16]. Lead ions can be absorbed by human body through respiratory and digestive systems, and most of lead ions will deposit in the body for a long time. It has been reported that the blood lead level in 24% of Chinese children exceeds 100μg/L, which could result in lead poisoning [17, 18]. These data indicate that China is facing a serious risk of lead poisoning. Notably, in mainland China, many industrial factories are established in low-income areas. Although the environmental awareness of people in these areas has gradually increased in recent years, lead pollution incidents have also frequently occurred, resulting in high concentration of Pb^2+^ in the environmental water [19]. Moreover, a previous study has reported that three kinds of MWCNTs possessed obvious lower adsorption rates of Pb^2+^ in the high concentration of salts than that in the low concentration of salts [20]. Therefore, we think that although the daily drinking water for people in low-income communities has been purified using MWCNTs, they may also have more opportunity for co-exposure to MWCNT and lead ions. Thus far, potential hepatotoxicity caused by MWCNTs has been investigated, while the further influences of MWCNTs on NAFLD patients and the hepatotoxicity of co-exposure to MWCNTs and lead ions have not yet been reported.

In the present study, we systematically investigated the toxicity of MWCNTs, lead acetate (PbAc), and co-treatment with them to the healthy and NAFLD mice. No significant changes in indicators including organ index, TG, HDL, LDL, and CHOL were observed in both the healthy and NAFLD mice after treatments with PbAc, MWCNTs or MWCNTs + PbAc. However, obvious liver function injury was observed only in NAFLD mice. Furthermore, MWCNTs and PbAc exposure significantly aggravated the nonalcoholic steatohepatitis phenotype, hepatic fibrosis and steatosis in NAFLD mice. Apoptotic analyses also demonstrated that MWCNTs and PbAc exposure significantly induced apoptosis in primary hepatocytes isolated from NAFLD mice. Meanwhile, co-treatment with MWCNTs and PbAc also led to hepatic lipid peroxidation, and significantly increased the expression levels of inflammatory cytokines in NAFLD mice livers.

These findings indicated that the low dose of MWCNTs and PbAc used in our study could significantly aggravate hepatic damages in NAFLD mice, but had no obvious hepatotoxic effects on healthy mice. Therefore, we suggest that NAFLD mice are more vulnerable to co-exposure to MWCNTs and PbAc compared with healthy mice. Low immunity and antioxidant capacity of NAFLD mice might explain the results. Our results also suggest that the NAFLD population should pay more attentions to improve the quality of their daily drinking water.

In our study, we also demonstrated that co-treatment with MWCNTs and PbAc may elicit their hepatotoxicity to NAFLD mice via inhibiting the AMPK/PPAR-γ pathway. It has been reported that PPAR-γ can activate hepatic stellate cells and further accelerate the hepatic recovery from chronic damage [21]. Meanwhile, PPAR-γ activation can promote the formation of monounsaturated fatty acids and inhibit oxidative stress [22]. It has also been reported that AMPK/Sterol-regulatory element binding proteins1 (SREBP1) pathway and its downstream targets are closely linked with lipid metabolism and oxidative stress [23], and AMPK can coordinate the long-term adaptation of lipid metabolism by suppressing SREBP1 [24].

Thus far, the hepatotoxicity of MWCNTs or lead ions has been well investigated [8, 25]. NAFLD has also been known to be a high risk for liver injury [26]. In the current study, we tried to explore the hepatotoxicity of co-exposure to MWCNTs and PbAc to NAFLD mice. Hence, we think that lack of significant novelty is a limitation of our present study. Moreover, further research aiming to explore the influence of MWCNTs and PbAc on the downstream targets of AMPK/PPAR-γ pathway, such as fatty acid synthase (FAS) and acetyl-CoA carboxylase (ACC) should be performed.

In summary, this research demonstrated that NAFLD mice were more vulnerable to MWCNTs and PbAc than healthy mice. Co-exposure to MWCNTs and PbAc significantly aggravated the hepatic damages in NAFLD mice. Our research could provide a biosafety evaluation for the application of MWCNTs in wastewater treatment.

## MATERIALS AND METHODS

### Animal models

Male C57BL/6J mice (four-week-old) were purchased from Charles River Laboratories (Beijing, China) and housed in light-controlled (12-h light/dark cycle) cages with free access to food and water. All experiments were approved by the Institutional Animal Care and Use Committee of Linyi people's hospital. 185 mice were randomly separated into the control group (n=85) and the NAFLD group (n=100). Mice in the NAFLD group received a high-fat diet, while mice in the control group received a normal diet. Body weight of each mouse was recorded every week. After 8 weeks, five mice in each group were sacrificed and their organs including heart, liver, lung, spleen and kidney were excised. After being washed with PBS for three times, the weight of such organs was measured.

80 mice in each group were randomly divided into 8 groups, respectively. Mice were fed intragastrically with saline water or various doses of multi-walled carbon nanotubes (MWCNTs), lead acetate (PbAc) or MWCNTs + PbAc every day. Both MWCNTs and PbAc were sterilized by high temperature and high pressure. The details were list in the Table 1. For further studies, 15 NAFLD mice were randomly assigned into 3 groups. Upon the administration of MWCNTs + PbAc, NAFLD mice were orally dosed with PF-06409577 (100 mg/kg) or simultaneously injected with T0070907 (10 mg/kg, intraperitoneally). NAFLD mice received oral and intraperitoneal administration of vehicle (DMSO) were considered the control. Body weight of each mouse was measured every week. After 2 weeks, all mice were sacrificed and the weight of their organs including heart, liver, lung, spleen and kidney was measured as above described. Organ index was calculated according to the equation: organ index=organ weight/body weight. After weighing, livers were immediately frozen in liquid nitrogen or fixed with 4% paraformaldehyde for further studies. All blood samples used in our study were collected from mouse hearts and placed into tubes containing EDTA as an anticoagulant. Plasma was separated within 30 min at 4°C and subsequently stored at −80°C for further researches.

**Table 1:** Information of animal experiments.

**Mice**	**Grouping**	**High dose**	**Low dose**
Control mice	control	saline water	saline water
	PbAc	300 mg/kg	150 mg/kg
	MWCNT	30 mg/kg	10 mg/kg
	MWCNT+ PbAc	30 mg/kg +300 mg/kg	10 mg/kg +150 mg/kg
NAFLD mice	control	saline water	saline water
	PbAc	300 mg/kg	150 mg/kg
	MWCNT	30 mg/kg	10 mg/kg
	MWCNT+ PbAc	30 mg/kg +300 mg/kg	10 mg/kg +150 mg/kg

### MWCNTs characteristics

Carboxyl MWCNTs (purity > 95%, diameter: 5–15 nm, length: 0.5-2 μm, COOH content 5.58 wt%) were purchased from Nanjing XFNANO Materials Tech Co., Ltd., China. As shown in Supplementary Figure 1, the morphology and size of MWCNTs were detected using a transmission electron microscope (TEM).

### Measurement of lipoprotein and hepatic damage biomarkers

According to the manufacturer’s instructions, the serum levels of CHOL, TG, HDL, LDL, ALT, AST and ALP were detected by an automatic biochemical analyzer (Beckman Coulter, Shanghai, China) with corresponding kits (Beckman Coulter).

### Histological analysis

Liver specimens were fixed in 4% paraformaldehyde, dehydrated, embedded in paraffin, cut into 4-μm sections, and subsequently stained with hematoxylin and eosin (H&E) as described previously [27]. Then the sections were viewed and photographed under an optical microscope (Olympus, Tokyo, Japan). Scoring of liver damage was performed in a blinded manner as previously described [11].

### Masson staining

Masson staining was performed as previously described [28]. Briefly, after being dewaxed, the liver sections were soaked in potassium dichromate overnight and washed with tap water. Subsequently, sections were dyed with hematoxylin for 3 min and then stained with ponceaux for 10 min. Finally, sections were dyed with phosphomolybdic acid solution and aniline blue solution for 3 min, respectively. After being sealed with neutral gum, all sections were visualized and photographed under an optical microscope (Olympus).

### Oil red O staining

As previously described [29], fresh frozen liver tissues were cut into 8-μm sections for Oil red O staining. Sections were fixed in ice-cold 4% paraformaldehyde for 20 minutes and washed thrice with distilled water. Then, sections were placed in propylene glycol for 5 minutes and stained with 0.5% oil red O solution for 8 minutes at 60°C. Subsequently, sections were rinsed in 85% propylene glycol solution for 30 min, washed with distilled water and dyed in hematoxylin solution for 30 s. Finally, all sections were immobilized with neutral gum and examined under an optical microscope (Olympus).

### Primary hepatocytes isolation, culture and treatments

Primary hepatocytes were isolated using a two-step collagenase perfusion method as previously described [30]. Primary hepatocytes were cultured in William’s E medium (Gibco, NY, USA) supplemented with 10% fetal bovine serum (FBS, Gibco), 2 mM L-glutamine (Sigma, MO, USA), 0.1 μM insulin (Sigma), 0.1 μM dexamethasone (Sigma) and 1% penicillin/streptomycin (Beyotime, Shanghai, China). Primary hepatocytes were subsequently incubated with PbAc (150 μg/mL), MWCNTs (10 μg/mL) or both of them, and intervened with PF-06409577 (0.5 μM), A-769662 (10 μM) or T0070907 (50 μM) for 24 h (compounds were all purchased from Sigma). Sterile saline water or dimethyl sulfoxide (DMSO) was administrated as the control. Finally, the treated primary hepatocytes were used for further apoptotic analysis, western blot analysis and cell viability assay.

### Apoptotic analysis

After being treated, primary hepatocytes were collected, washed with PBS twice, and incubated with 10 μL Annexin-V-fluorescein isothiocyanate (FITC) and 5 μL PI (both were purchased from Sangon Biotech Co., Shanghai, China) at room temperature in the dark for 15 min. Then, apoptotic analyses were performed with a flow cytometer (BD Biosciences, California, USA).

### Assessment of liver TG, oxidative stress biomarkers and inflammatory cytokines

As previously described [8, 31], the mouse livers were homogenized in 50 mM Tris-HCl buffer (pH 7.4) containing 1.15% potassium chloride. Then, the homogenate was centrifuged at 12,000 g for 15 min at 4 °C and the supernatant was collected for further biochemical assays. Hepatic protein concentrations were measured with a Bradford kit (Bio-Rad, Hercules, USA). The levels of liver TG, GSH, H_2_O_2_, MDA and the activities of GPx, GST, SOD were assayed by commercially available kits according to the manufacturer’s instructions (all kits were purchased from Solarbio, Beijing, China; TG kit lot: BC0620, GSH kit lot: BC1175, GPx kit lot: BC1190, GST kit lot: BC0350, H_2_O_2_ kit lot: BC3595, SOD kit lot: BC0170, MDA kit lot: BC0025). The concentrations of inflammatory cytokines interleukin-6 (IL-6), interleukin-1β (IL-1β) and tumor necrosis factor alpha (TNF-α) were measured using commercially available ELISA kits (Abcam, Cambridge, UK, ab100713, ab197742, ab208348) according to the manufacturer’s instructions.

### Western blot analysis

The protein extraction and western blotting were conducted as described previously [32, 33]. Briefly, the mouse livers or primary hepatocytes were lysed in RIPA lysis buffer (Beyotime) and protein concentrations were measured with a Bradford kit (Bio-Rad, Hercules, USA). Then, the protein (50 μg) was loaded and separated by 8% sodium dodecyl sulfate polyacrylamide gel electrophoresis (SDS-PAGE) gels and transferred to polyvinylidene fluoride (PVDF) membranes (Merck Millipore, Darmstadt, Germany). Membranes were then blocked in Tris buffered saline containing 5% skim milk at room temperature for 2 h and incubated overnight at 4 °C with various primary antibodies as follows: anti-PPARγ (Abcam, Cambridge, UK, ab59256, 1:500), anti-p-AMPKα (Cell Signaling Technology, Inc., Danvers, MA, USA, #2537, 1:1000) and anti-GAPDH (Beyotime, AG019, 1:1000). Membranes were subsequently incubated with horseradish peroxidase-conjugated secondary antibodies (Beyotime, A0208, A0216, 1:5000) at room temperature in 5% skim milk for 2 h. The protein bands were visualized with a LAS-3000 luminescent image analyzer (Fujifilm, Tokyo, Japan) and quantified using the Quantity one software (Bio-Rad laboratories, Inc. Hercules, USA).

### Cell viability assay

After being treated, the cell viability of primary hepatocytes was measured by the Cell Counting Kit-8 (CCK-8, Dojindo Laboratories, Kyushu, Japan) according to the manufacturer's instructions. The optical density (OD) values at 450 nm were recorded on a microplate reader (Bio-Rad).

### Statistical analysis

Data are presented as the mean ± S.D. Statistical analysis was performed with SPSS version 13.0 (SPSS Inc., Chicago, USA). Significant difference between each group was analyzed using unpaired 2-tailed Student’s t tests or one-way ANOVA. A *p* value < 0.05 was considered statistically significant.

## Supplementary Material

Supplementary Figures
