# Supplementary material for: Co-exposure to multi-walled carbon nanotube and lead ions aggravates hepatotoxicity of nonalcoholic fatty liver via inhibiting AMPK/PPARγ pathway
Source: Aging (Albany NY). 2020 Jul 17;12(14):14189–204. doi: 10.18632/aging.103430 (PMC7425511; doi:10.18632/aging.103430)
Supplement: Supplementary Figures [file aging-12-103430-s001..pdf]

SUPPLEMENTARY FIGURES

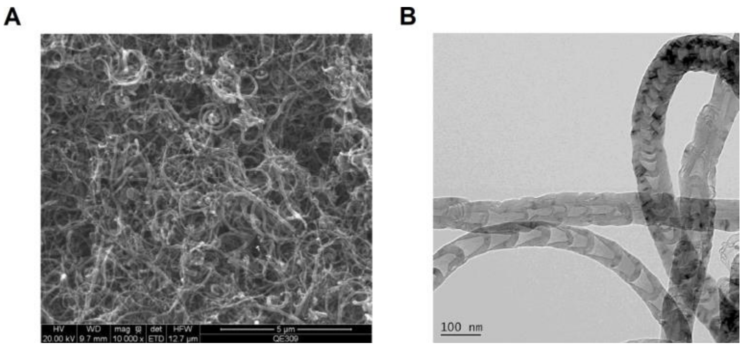

Supplementary Figure 1. TEM photographs of carboxyl MWCNTs. (**A**) a low magnification (×10000, 5 μm) and (**B**) a high magnification (×50000, 100 nm).

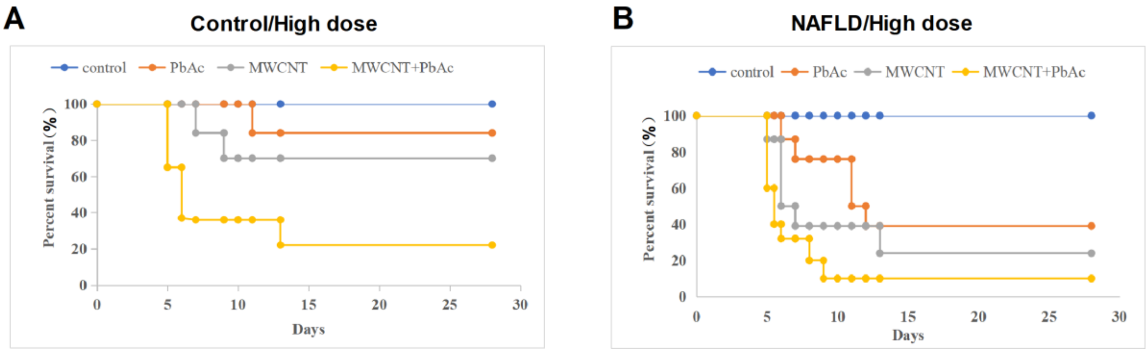

Supplementary Figure 2. The survival curve of the control and NAFLD mice exposed to the high dose of PbAc, MWCNTs or MWCNTs + PbAc.
